# A Survey of Partition-Based Techniques for Copy-Move Forgery Detection

**DOI:** 10.1155/2014/975456

**Published:** 2014-07-22

**Authors:** Wandji Nanda Nathalie Diane, Sun Xingming, Fah Kue Moise

**Affiliations:** ^1^School of Information Science and Engineering, Hunan University, Changsha 410082, China; ^2^Jiangsu Engineering Center of Network Monitoring, Nanjing University of Information Science and Technology, Nanjing 210044, China; ^3^The University of Yaoundé I, Yaoundé, Cameroon

## Abstract

A copy-move forged image results from a specific type of image tampering procedure carried out by copying a part of an image and pasting it on one or more parts of the same image generally to maliciously hide unwanted objects/regions or clone an object. Therefore, detecting such forgeries mainly consists in devising ways of exposing identical or relatively similar areas in images. This survey attempts to cover existing partition-based copy-move forgery detection techniques.

## 1. Introduction

**Figure 1:**
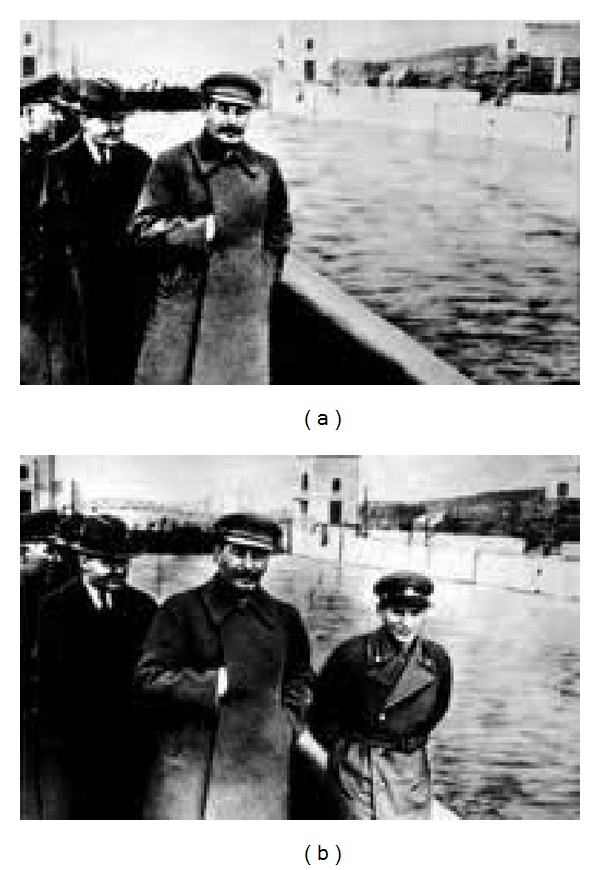
Stalin had his enemies concealed.

Copy-move forged images have a long history, going back to the early 1900s, when the Soviet dictator Joseph Stalin successfully had his enemies concealed in photographs [1] (Figure 1). Although possible, this task was both extremely difficult and time-consuming because of the limited devices and tools available in that era.

**Figure 2:**
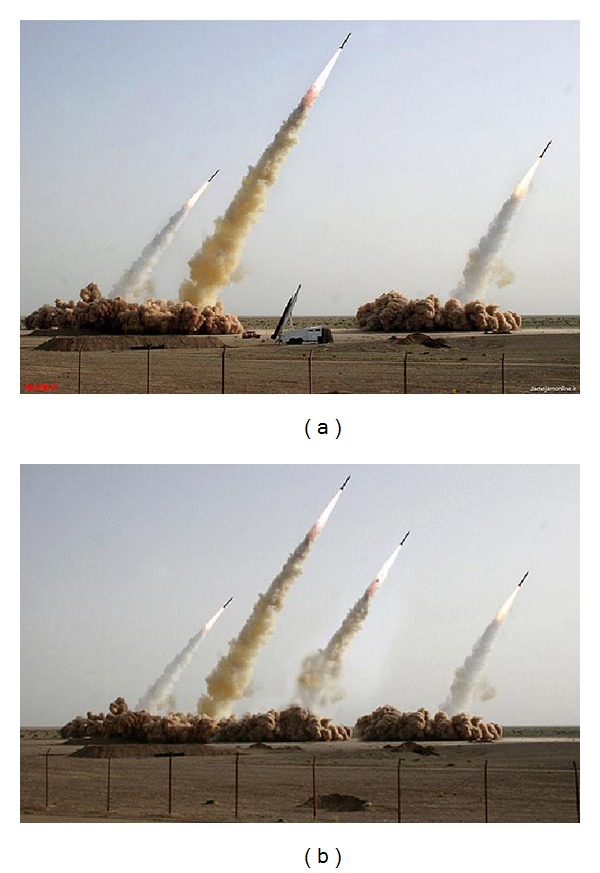
(a) Original photograph. (b) Forged photograph released by Iran.

Contrary to this, nowadays, little or no effort is needed to perform such task. This is due to the numerous affordable, powerful, and extremely easy-to-use digital image acquisition, processing, and editing devices and tools at the disposal of the end users, regardless of their professionalism. Therefore, both professionals and amateurs can now, with no major difficulty and rapidly, alter images without leaving any visual clue. This situation, coupled with the widespread transmission through the Internet, has raised a crucial and challenging problem that can be summed up in this intriguing question: is seeing believing? In other words, the society/public has lost its confidence in digital images, especially when it comes to sensitive data such as medical records, news items, evidence in court of law, and political propaganda, to name a few. Among the various copy-move forgery related incidents that had a great impact on the trust the general public has in digital photographs, we can mention the altered photograph released by Iran (Figure 2) on July 9, 2008, and published by several western media, including The New York Times and The Los Angeles Times, showing four missiles rising into the air instead of three during a test firing at an undisclosed location in the Iranian desert.

To tackle this crisis of confidence and attempt to restore the credibility in society regarding digital images, the field of digital image forensics came up with the primary aim of developing efficient and reliable image forgery detection methods.

Currently, several techniques have been proposed for the exposure of image forgeries in general, and especially copy-move forgeries. The existing copy-move forgery detection techniques are based on the fact that, at the end of the manipulation process, the resulting image will have relatively similar areas since the duplicated regions come from the same image. Although not always necessary, some additional postprocessing operations are often performed either on the copied region before pasting it or on the whole forged image, to make the forgery unnoticeable. Among them, we find noise addition, rotation, translation, scaling, lossy compression, blurring, flipping, edge sharpening, change of intensity, reflection, and contrast changes. Therefore, research in this field aims at detecting duplicated image regions, even if they are slightly different from each other and will consequently consist in finding wide relatively identical areas in a given image. The easiest way to detect such forgery is trivially the exhaustive search, but this approach has two major drawbacks: firstly it is computationally costly for large images since it takes *MN* ^2^ steps for an image of size *M* × *N* and secondly it fails to detect the forgery in case the copied portion has undergone some modifications. A second and more efficient approach, which was proposed by Fridrich et al. in [2], is the use of autocorrelation properties. Nevertheless, this approach was shown to fail in case the duplicated region is not a large portion of the original image.

Recently, research has taken a partition-based approach, focusing on partitioning the image and matching partitions or features extracted from partitions rather than pixels. A partition can be a rectangular block, a circular block, an object in the image, or a subimage (an image being divided into overlapping/nonoverlapping subimages of equal size). Partition-based approaches start by dividing the suspected image into overlapping/nonoverlapping partitions. This division is often followed by the extraction of robust features from each partition in order to make the detection faster and more efficient even in case of further processing of the copied region. At last, the obtained features are sorted or arranged using appropriate data structures to make a sufficiently reliable decision based on the similarity of neighboring pairs. It is worth noting that the image can eventually be preprocessed before the feature extraction procedure is carried out and the result of the detection procedure can also eventually be postprocessed to eliminate isolated points by grouping matches that are collectively conformed to a certain pattern.

There are already some attempts to review copy-move forgery detection techniques, like those reported in [3–7] but most of these existing surveys mainly focus on specific steps of the detection procedure and are not exhaustive. To the best of our knowledge, this survey is the most complete in the field of partition-based copy-move forgery detection approaches. Based on the foregoing, the latter approaches can be divided into two main classes depending upon the partitioning plan. We will classify as* block-based approaches* the approaches that uniformly partition the image into small overlapping/nonoverlapping rectangular or circular partitions called “blocks” of fixed size and as* nonblock-based approaches* any partition-based approach that does not fall into the first category. The rest of this survey is organized as follows. Sections 2 and 3 are both dedicated to the survey of partition-based approaches.  Section 2 will focus on block-based techniques, whereas Section 3 will focus on nonblock-based techniques.  Section 4 provides the conclusion remarks.

## 2. Block-Based Approaches

**Figure 3:**
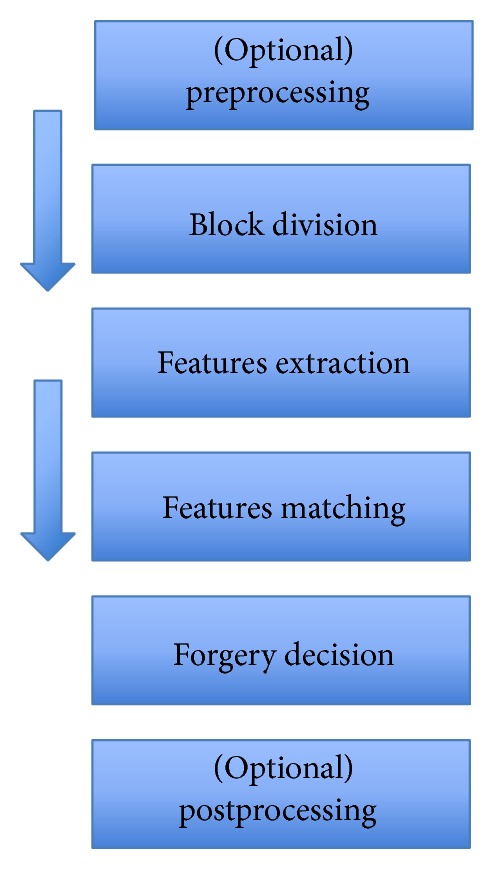
Common pipeline for block-based detection of copy-move forgeries.

Most of the block-based approaches follow the pipeline described in Figure 3. A detailed description of each step of the block-based detection procedure is given in what follows.

### 2.1. Preprocessing

Among the existing preprocessing techniques, the most used is the* color conversion*. In fact, many existing detection methods [8–34] require a merger of the red, green, and blue color channels since they operate on gray scale images. Instead of the grayscale conversion, some methods require the image to be first converted from the RGB (Red, Green, and Blue) color system to the YCbCr color system since they operate only either on the luma(Y) information [35–38] or on one of the chrominance components [39]. Some other methods combine the use of the RGB color information and the luma information as seen in [40–42] or the use of the RGB color information and the gray-level information as in [43].

Apart from the color conversion approach, authors in [44–48] used a* discrete wavelet transform (DWT) based dimension-reduction* preprocessing technique. The latter technique works by applying DWT to the input image and extracting the resulting low-frequency subband that will be used in the remaining steps of the detection procedure.

Other techniques such as the* image resizing* into a specific scale to reduce the time needed for the detection in [28, 35, 38], the* low-pass filtering* in [15, 23, 35, 49]; the* Gaussian pyramid* in [48, 50–52], the* degraded palette* obtained by decreasing the image details in [53], the* integral image* in [24], and the* conversion to 8-bit gray level* followed by a* mean filter kernel* in [54] also exist in the literature.

### 2.2. Partitioning

Due to the fact that in a copy-move forgery, the source and the target regions are both located in the same image; the forged image must exhibit at least two similar regions. This will rarely be the case for a natural image, except for images having two large smooth regions. Thus the detection method will mainly search for large similar regions in a given image. To avoid the high computational cost of the exhaustive search, the image is first divided into blocks and the comparison is done at a block level. The blocks are either square or circular.

- Square blocks partitioning: this is the most common type of partitioning and it is used by authors in [9–14, 28–35, 53–75]. The image is divided into overlapping/nonoverlapping square blocks by sliding once along the image a window of size *b* × *b* from the upper left corner right down to the lower right corner.
- Circular blocks partitioning: instead of partitioning the image into square blocks, some authors used the corresponding inscribed discs as seen in [42, 52].

**Figure 4:**
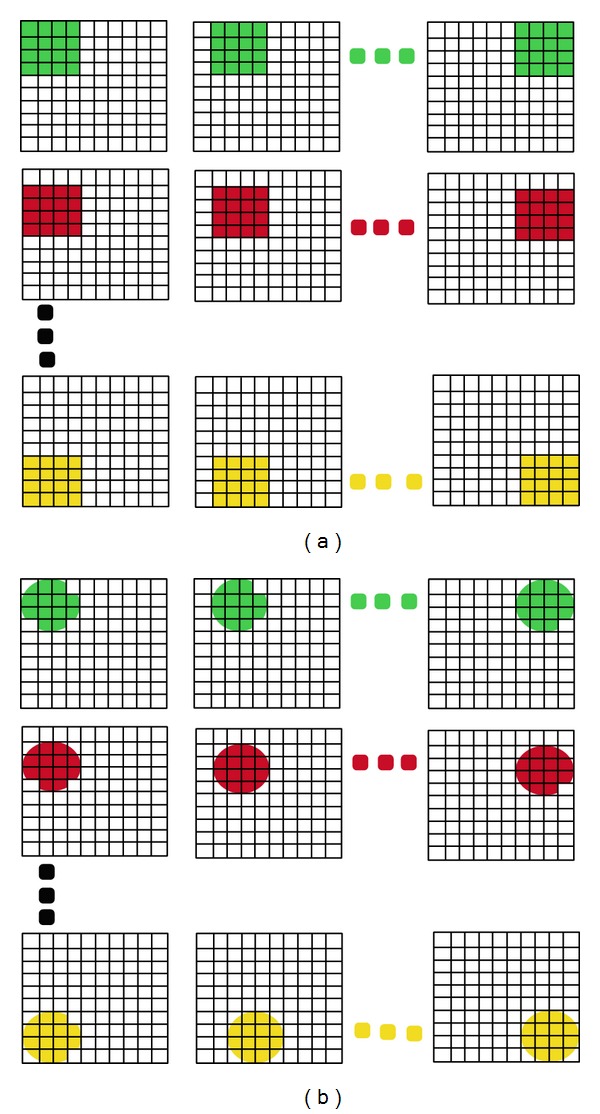
Overlapping square 4 × 4 block division and corresponding overlapping circular block division.

The partitioning into overlapping blocks is illustrated in Figure 4.

**Figure 5:**
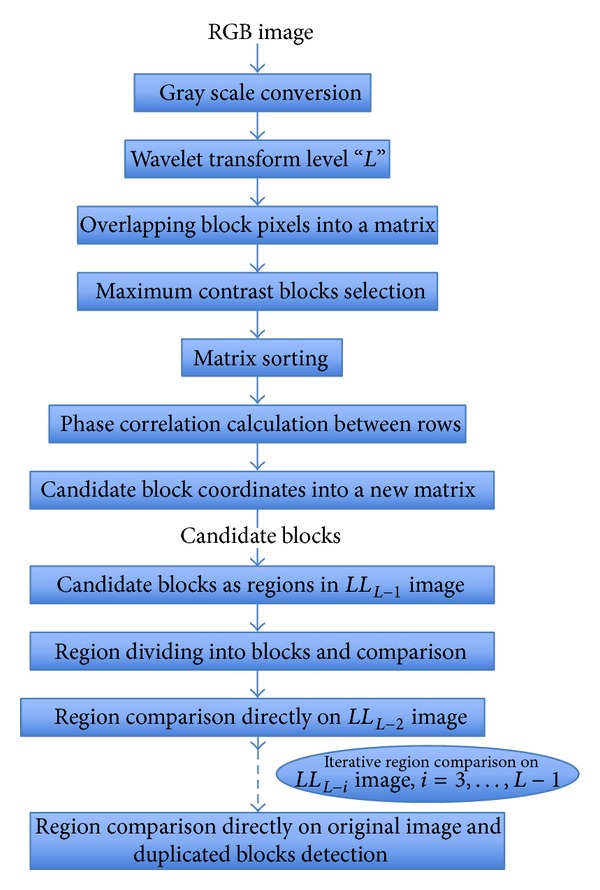
Flowchart of the method proposed by Khan and Kulkarni in [46].

In the literature, there are few methods that operate directly on blocks without feature extraction. This is the case, for example, in [49, 76]. It is also the case in [46] (Figure 5) where the comparison is iteratively done at each level *k* of DWT decomposition on the corresponding low-frequency subband LL_*k*_. Because of the high dimensionality of blocks, such an approach calls for some dimensionality reduction that is achieved through the use of a locally linear embedding (LLE) in [62] and principal component analysis (PCA) in [68]. Nevertheless, for a large majority of the existing copy-move forgery detection techniques, feature extraction is a prerequisite step.

### 2.3. Feature Extraction

The detection accuracy mainly depends on the ability to map the blocks in a copy-move pair to similar features even in case of postprocessing. To accomplish this goal, many procedures have been proposed.

**Figure 6:**
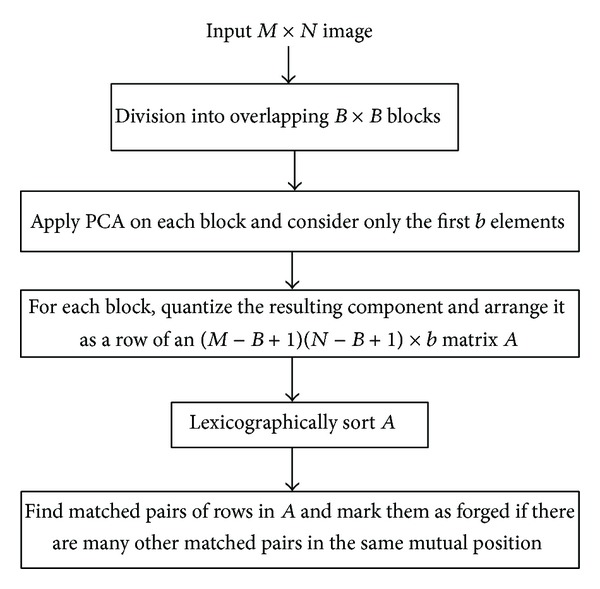
Flowchart of the method proposed by Popescu and Farid in [68].

*Dimensionality Reduction-Based.* In [68] (Figure 6), blocks are reduced in dimension by applying PCA algorithm and the quantized resulting components are arranged as rows of a matrix. This method, based on PCA, is robust to noise addition and lossy compression. It was improved in [62] by a new technique based upon LLE (locally linear embedding). The latter method works in a way similar to [77]. Later, authors in [77] proposed to compute the improved circular projection vector for each block. The matrix constituted by all the obtained circular projection vectors was then reduced in terms of its dimensionality by applying PCA and retaining only components with a high cumulative contribution. The proposed technique was proven to be robust for rotation of an angle not greater than 60 degrees. In [29], for each block, kernel PCA- (KPCA-) based vectors and DWT vectors are computed. Obtained KPCA-based vectors are robust to noise addition and lossy JPEG compression. 

*Multiscale Autoconvolution Invariants-Based.* In [16], multiscale autoconvolution (MSA) invariants are extracted for each block yielding a scheme robust to rotation, Gaussian noise addition, and JPEG compression. 

**Figure 7:**
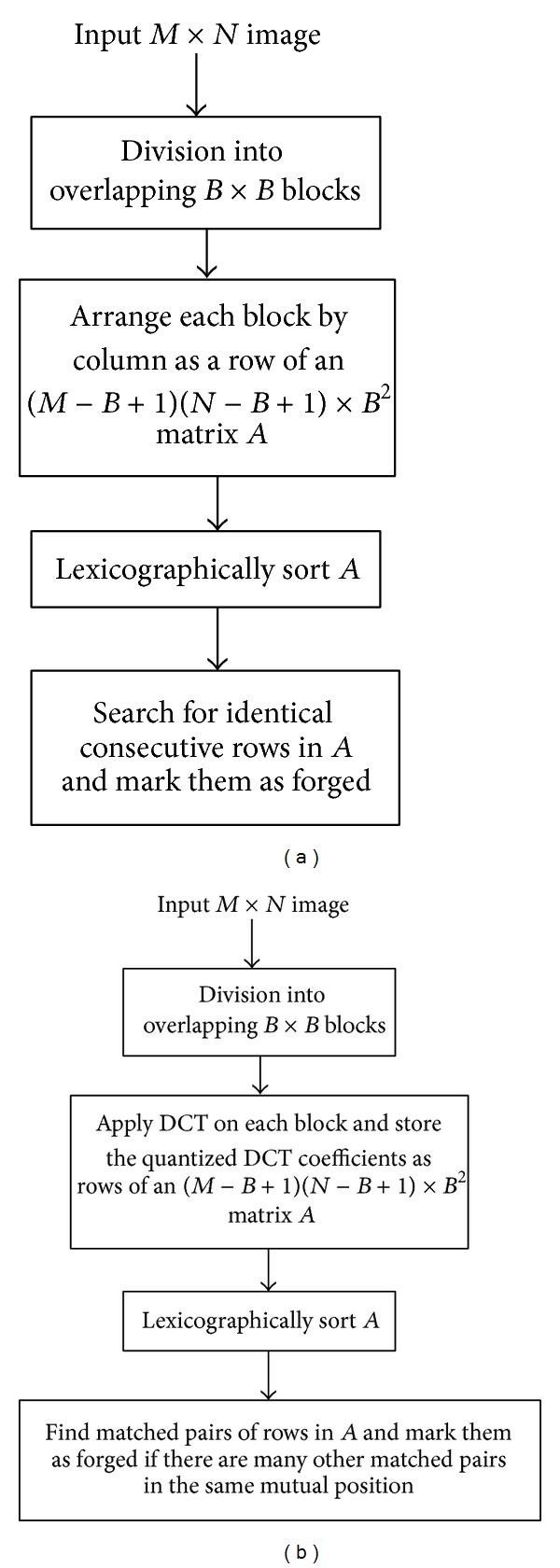
Flowchart of block-based methods proposed by Fridrich et al. in [2].

*Discrete Cosine Transform (DCT)-Based.* In [2] (Figure 7(b)), DCT coefficients are computed for each block and then quantized to be used as the block's representation. Authors in [47] proposed the same blocks representation. the only difference with [2] is that, here, the blocks are extracted from the low-frequency subband of the discrete wavelet transformed image and not from the whole image leading to a smaller number of blocks to process. In [70], the quantized values are arranged in zigzag order in a 1D array. Then, truncating the obtained array yielded a reduced dimension representation of the features. The method has been proven to be robust to JPEG compression and blurring. In [57], according to their frequency, 8 coefficients are selected from the array constituted by the quantized coefficients in zigzag order. The method is robust to blurring and noise contamination. As for authors in [27], after the quantized coefficients are reshaped to a 1D array in zigzag order for each block, the low-frequency coefficients are down-sampled to make the DC component close to zero and the dimensionality is reduced by the PCA algorithm applied on the array. In [10], each discrete cosine transformed block is represented by its inscribed disc block. Following, the circular block is divided into 4 equal parts and a feature vector of length 4 is extracted by computing the mean values of each part. The method is robust to Gaussian blurring and noise contamination. In [40], for each block, DCT is applied on each of the three color channels R, G, and B (RGB color system) and also on the luma-component *Y* (YUV color system). A feature vector of length 9 mainly depending on DCT coefficients and average intensity values of each channel is computed. The method is relatively robust to some common distortions such as JPEG compression, blurring, and Gaussian noise contamination. In [31], each 2D-DCT quantized block is divided into nonoverlapping square subblocks and the largest singular value of each subblock is computed by applying SVD to it. The obtained subblocks largest singular values are combined to form the feature vector representing the corresponding block. The method is robust to JPEG compression, AWGN, and Gaussian blurring. In [56], DCT is applied to each nonoverlapping square block and the first 8 elements of the upper left corner of the resultant matrix M of size 3 × 3 (read row-wise) are extracted to form a vector; that is, *B*_*k*_ = (*m*_11_, *m*_12_, *m*_13_, *m*_21_, *m*_22_, *m*_23_, *m*_31_, *m*_32_). These vectors are stored row-wise in a matrix *B*. On the other hand, DWT is applied to each block Bloc (*i*, *j*) and the low-frequency subband LL_*k*_ is extracted. For each block, the feature vector *F*_*k*_ is obtained by *F*_*k*_ = *B*_*k*_∗LL_*k*_. These features were proven to be robust to JPEG compression with a quality factor greater or equal to 20 and noise addition. 

*Wavelet-Based.* In [33, 34], 2D-DWT is applied on each block at level 2. In [33], at each level, 5 features are extracted from each of the 4 obtained subbands as mean, 1-norm, 2-norm, standard deviation, and average residual. As to the method in [34], the components of the 7 resulting subbands are quantized to form the blocks feature vector. In [58], two orthogonal wavelet transforms are applied on each block, namely, the discrete cosine transform wavelet (DCTW) and the Walsh wavelet (WW). Further, for each block, a feature vector is built by computing the mean, the sum of the absolute values, the square root of the squared values' sum, the standard deviation, and the average residual of the pixels in the block. The method is robust to blurring and edge sharpening operations. 

*Log Polar Mapping-Based.* In [25], each block in the low-frequency subband is mapped from rectangular coordinates into log-polar coordinates. This is iteratively done at each wavelet decomposition level. The method is robust to translation and rotation. Authors in [42] solved rotation, reflection, and scaling by mapping each block to log-polar coordinates and then adding it up along the angle axis, therefore producing a one-dimensional (1D) robust descriptor. 

*Polar Harmonic Transform-Based.* In [19], features are extracted from each circular region by applying on it a polar harmonic transform (PHT). The method is robust to rotation, JPEG compression, and noise addition. As for [23], the polar sine transform (PST) is applied on each block to extract features that are affine invariant. The polar cosine transform (PCT) is applied on each block in [69] to extract rotation-invariant robust features. 

*Local Binary Pattern- (LBP-) Based.* In [21], each block is first filtered with an edge-preserving adaptive low-pass filter, for instance, the Wiener filter. Then, the multiresolution local binary patterns (MLBP) features are extracted from each block by combining the information provided by the use of multiple LBP operators on the blocks. The method is robust to scaling, JPEG compression, white Gaussian noise addition, and Gaussian blurring. As for [15], features are extracted from each circular block using uniform local binary patterns (LBP) which is rotation invariant. The method is robust to rotation, JPEG compression, white Gaussian noise addition, Gaussian blurring, and flipping. 

**Figure 8:**
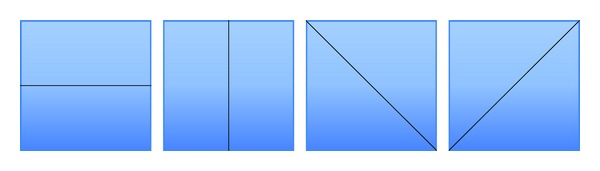
Block decomposition in [43].

**Figure 9:**
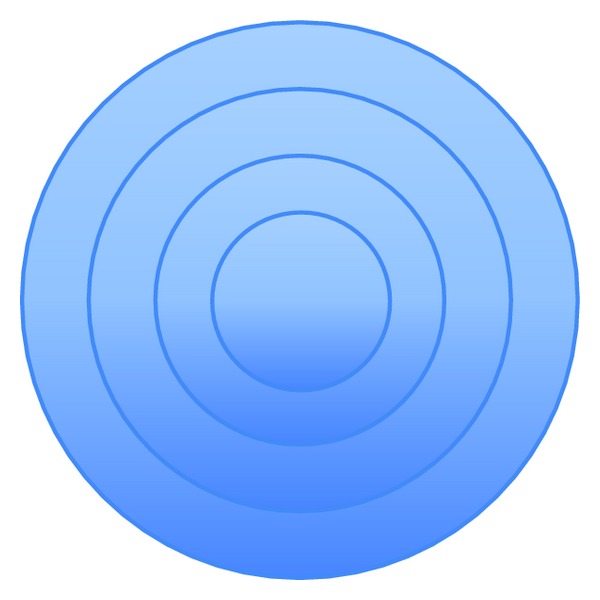
Block decomposition in [52].

**Figure 10:**
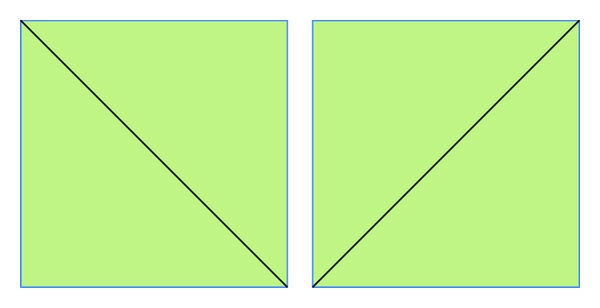
Block decomposition in [30].

*Texture-Based.* In [43], two sets of features were extracted. The first set of features included the average of red, blue, and green color components. As for the second set, in the Y channel, the blocks are divided into two parts, in 4 directions (Figure 8), and for each part, the ratio of the part's total intensity value over the block's total intensity value is calculated. The method was very robust to Gaussian blurring (5 × 5 window, sigma = 1), JPEG compression up to the quality level of 30, and additive noise with SNR = 24 dB. In [52], each circular region is divided into 4 concentric circles (Figure 9) and the mean pixel value in each region is used as feature, thus forming a feature vector of length 4. The method is robust to rotation, noise, blurring, and JPEG compression with a quality factor of 65. In [22], authors tested five texture descriptors, namely, the statistical descriptor (mean, standard deviation, skewness, and kurtosis of the pixels grey values); edge histogram; Tamura descriptors (contrast, coarseness, and directionality properties from the Tamura set of features); Gabor descriptors; and Haralick descriptors. They found out from experiments that the statistical descriptor gives the best results in terms of precision versus execution time. In [30], each block is divided for both diagonals yielding 4 subblocks (Figure 10). For each block, a 9-dimensional characteristic vector mainly depending on the average values of the block and each of its subblocks is computed. Authors in [42] proposed a method that proceeds as follows: for each block, the inscribed disc is taken and in that disc 4 features are computed: the average value of each of the three color channels, namely, red, green, and blue, on one hand and the entropy of the luminance of pixels within the disc on the other hand. A feature vector of length 4 that has the computed values as components will characterize each block. In [73], Gabor features are extracted from each block and stored in a matrix that is later reduced in dimension by using PCA algorithm. In [63], Gabor filter with different scaling factors, rotation angles, and frequencies is used to generate the Gabor feature representation of each block. Then, keypoints are extracted from the Gabor descriptor comparison and are used as Gabor features for the similarity check. Authors in [41] computed for each block and each RGB color channel the mean, the standard deviation, and the skewness yielding 9 values. Then, for the corresponding Y-component in the YUV color system, they computed the average value of pixels and concatenated all the obtained values to form a feature vector of length 10. The method is robust to noise addition. In [54], a color coherence vector of length 128 is computed for each block where the first 64 components represent the coherent pixel of the corresponding pixel value. On the contrary, the last 64 components represent the incoherent pixels. The method is robust to Gaussian blurring. Authors in [53] extracted color characteristics from each block and then clustered the obtained data by similarity of colors using the Hausdorff distance. 

*Curvelet Transform-Based.* In [9], the fast curvelet transform (FCT) of each block is computed and features are extracted as follows: for each subband of the fast curvelet transformed block, the mean values are calculated for each level of scale and arranged in a vector. The method is robust to scaling and rotation. 

*Fourier Transform-Based.* In [12], Fourier Mellin transform (FMT) is applied on the image blocks. For this purpose, the Fourier transform representation of each block is first obtained, and then the resulting magnitude values are resampled into log polar coordinates. A vector representation was obtained by projecting log-polar values onto 1D along radius direction and these representations were used as features. The method is robust to scaling by 10%, compression up to JPEG quality level 20 and slight rotation (less than 10 degrees). Authors in [75] used a similar approach as in [12] but instead of the radius direction, the log-polar values are projected onto 1D along angular direction and quantized to be used as features. The method is robust to rotation, slight scaling, and JPEG compression. In [36], log polar transform (LPT) is applied on each circular region followed by the Fourier transform (FT) of the log polar transformed region. The method is robust to rotation and scaling. In [28], the discrete Fourier transform (DFT) is applied on each block. In [61], 2D-FT is applied on each *b* × *b* block and the result is arranged in a vector of length *b* ^2^. The resulting vector is then quantized with a predefined value and its dimension is reduced by multiplying it by a constant in the range of [0,1]. The method is robust to blurring and JPEG compression attacks even if the quality factor is lower than 50. In [71], the log-polar fast Fourier transform (LPFFT) of each block is computed by first computing the fractional Fourier transform (FRFT) followed by grid conversion solely using 1D Fourier transform and interpolation. The method is tolerant to scaling and rotation. 

*Nonnegative Matrix Factorization- (NNMF-) Based.* In [35], nonnegative matrix factorization (NNMF) is applied on each block and the obtained coefficients are quantized. The method is robust to blurring. 

*Fast Walsh-Hadamard Transform- (FWHT-) Based.* Authors in [13] applied the FWHT on each block of the low-frequency subband to extract features. 

*Ridgelet Transform-Based.* In [32], the ridgelet transform is applied to each block and Hu moments obtained from the ridgelet transformed blocks are used to represent the block. The method is robust to JPEG compression. 

*Radon Transform- (RT-) Based.* In [64], Radon transform (RT) is applied on each block for a set of predefined angle values resulting in a matrix having the projections generated with defined angles as columns. The feature vector will be the resulting matrix arranged as a 1D array. 

*Dual Tree Complex Wavelet Transform- (DT-CWT-) Based.* In [26], the 3-level dual tree complex wavelet transform (DT-CWT) is performed on each block, and for each subband, channel energies are extracted. The feature vector consists of the magnitude of the discrete Fourier transformed energies. The method is robust to rotation. 

*Moment-Based.* In [65], 24 blur invariant moments are extracted from each block and this is done for each RGB color channel (Red, Green, and Blue). Therefore, a vector of length 72 represents each block and will be reduced in dimension by applying principal component analysis (PCA). These features were proved to be robust not only against blur but also against lossy JPEG compression, contrast changes, and noise addition in the forged area. The approach in [44] differs from the one in [65] only by the fact that blocks are extracted only from the low-frequency subband of the discrete wavelet transformed image and not from the whole input image. This leads to a reduction in time complexity but weakens the robustness against lossy compression. In [17], four merged blur and affine moment invariants are computed for each block and stored in a matrix. The method is robust to simple affine transform and blurring. Authors in [66] used Krawtchouk moments that are calculated for all blocks. The method is robust to white Gaussian noise addition and blurring. In [67], for each block, Zernike moments of precise order are computed and arranged in a vector. Blocks will be characterized by the magnitude values of the obtained vectors. The method is robust to rotation and noise contamination. In [74], they improved the block matching procedure in [67] by using a procedure based on locality sensitive hashing and for the feature space error-reduction procedure, the phase of Zernike moments was included. In [50], the feature vector has the sorted first four Hu moments extracted from the circular overlapping blocks as components. The method is robust to rotation, noise addition, blurring, and JPEG compression. In [51], for each block, a 1 × 7 sized feature vector is constituted by the exponenti-fourier moments on the one hand and the histogram invariant moments on the other. The method is robust to scaling, translation, and rotation. 

*Singular Value Decomposition- (SVD-) Based.* In [59], SVD is applied on each block and singular values feature vectors are extracted. In [11], the singular value decomposition is applied on overlapping blocks of the low-frequency component in wavelet subband. The difference with [59] is that the image is first preprocessed using DWT. These features are robust to compression up to JPEG quality level 70. Authors in [55] also used the same features as in [59]. The 2 methods only differ for the matching procedure. Authors in [60] applied an improved SVD on each block by selecting only the singular values of significance. The method is robust to noise addition. As for authors in [48], the difference with [11] is that the image is preprocessed using Gaussian pyramid before being discrete wavelet transformed to extract the low-frequency subband. 

*Key-Point Based.* In [38], the suspicious color image is first converted from RGB to YCbCr colorspace. The image is set to downsampling and divided into overlapping subblocks. Then, keypoints and corresponding descriptors are extracted both from the subblocks and the whole image using a approach based on SURF algorithm. Following, a matching procedure is applied on a matrix constituted by the extracted keypoints and feature descriptors.

### 2.4. Feature Matching

After the feature extraction, potential copy-move pairs are identified by searching the blocks with similar feature vectors. The extracted features are first arranged as rows of a matrix M. Then, the trivial approach is to compare each feature with all the other features but this is expensive in terms of computation time. To cope with this challenge, many methods exist to bring similar features close to each other, therefore reducing the computation time by suppressing useless comparisons. In fact, each feature will only be compared with a certain amount of its neighbors. Among the known methods, the most commonly used is lexicographic sorting [25–31] that consists of row-sorting *M* lexicographically to produce a resulting matrix having similar features adjacent to each other, and consequently making them easier to detect. In addition to lexicographic sorting, it is worthy to mention the radix sorting [14, 78], the sorting according to the number of zeroes [35], the k-dimensional tree (kd-tree) [21, 65], the combination of lexicographic sorting, and kd-tree used to improve both the time complexity and the accuracy in the feature matching step [21], the counting of blooms filters [12], the sorting according to vectors component which has the maximum variance along all the features [22], the hash values comparison and blocks linking [75], and the blocks clustering [79].

Once the data is organized so as to reduce the complexity of the similarity check, the search of similar features is done using various similarity criteria amongst which can be mentioned the Euclidean distance [50–52]; the measure *S* = 1/(1 + dis) [32, 44] where dis is a distance measured in the Euclidean space; the Hamming distance [35]; the Hausdorff distance [53]; the logical distance [30]; the correlation coefficient [17, 42]; the phase-correlation [46, 64]; the normalized cross-spectrum [36, 71]; the local sensitive hashing [69, 74]; the ratio of the absolute error and the minimum value of the two components [22]; the mean and variance of the difference vector diff (diff(*u*, *v*) = (*u*_1_ − *v*_1_, *u*_2_ − *v*_2_,…, *u*_*n*_ − *v*_*n*_) where *u* = (*u*_1_, *u*_2_,…, *u*_*n*_) and *v* = (*v*_1_, *v*_2_,…, *v*_*n*_)) [56]; the absolute value of each component of the difference vector combined with their partial sums [43]; the absolute value of each component of the difference vector combined with the ratio of these absolute values and the minimum of the corresponding feature vector components taken as absolute values [33]; the sum of absolute values of components of the difference vector [54]; the absolute value of the difference between their block numbers (position of the top-left corner point) [13]; and the element by element comparison [61].

### 2.5. Forgery Decision

Almost always, the single similarity criterion is not enough to decide on the presence/absence of duplicated area. This is due to the fact that most natural images might contain one or more pairs of highly similar regions, consequently yielding false matches. Therefore, there is a need to identify characteristics of copy-moved regions to distinguish them from falsely matched ones. In the literature, different algorithms are used for instance:

- the same affine transformation selection (SATS) in [78, 80],
- the multihop jump (MHJ) algorithm in [13],
- the difference (shift vector) between the coordinates of every matched pair of feature vectors (adjacent pairs in case of lexicographic sorting) is computed in [29–34]: it is supposed that all the copy-move pairs must have the same displacement vector. The accumulated number of each of the shift vectors is then computed and the large accumulated number is considered as eventual presence of a duplicated region,
- the distance criterion: the spatially adjacent blocks in an image are likely to be identified as similar. Therefore, a potential pair composed of two spatially adjacent blocks should be considered as a false match. To identify such pairs, for each matched pair, three measures are used: the euclidean distance between the corresponding blocks' spatial location in [64–67]; the max-norm distance in [11, 16], and the distance ratio of the closest to the second closest neighbor in [59]. A pair is discarded if the computed distance is smaller than a certain threshold, meaning the blocks are close to each other in the image,
- the area criterion: let A1 and A2 be the two regions obtained from the matching procedure; the algorithm in [19, 43, 52] determines whether a copy-move tampering has occurred based on two values: *v*_1_ = min⁡⁡(|*A*1 | , |*A*2|) and *v*_2_ = ||*A*1| − |*A*2||/max⁡⁡(|*A*1 | , |*A*2|). If *v*_1_ > *αM*∗*N*∗0.85% and *v*_2_ < *T* where *α* and *T* are preset thresholds, then *A*1 and *A*2 are marked as copy-move regions.

### 2.6. Postprocessing

Sometimes, the duplicated regions map obtained from the previous step needs to be further processed. This can be done, among others, by morphological postprocessing as suggested in [10, 27] which include opening, erosion, and dilatation operations; a sliding window as in [15, 23]; a square kernel mean filter as in [61]; and the RANdom SAmple consensus (RANSAC) algorithm as in [21] that identifies inliers and removes outliers.

## 3. Nonblock-Based Approaches

### 3.1. Segment-Based Approaches

One approach to identifying copy-move forgeries involves partitioning the image into segments so that a single segment fully contains an object, and the segment is relatively homogeneous. In [81], the noise image *I*_*n*_ of the input image *I* is computed and for each segment of *I*; the corresponding noise segment *S*_*n*_ is extracted from *I*_*n*_. Finally, histograms of the extracted noise segments *S*_*n*_ are compared for similarity check. As for [82], authors proposed to apply the dyadic wavelet transform (DyWT) to the input image at scale 2. For the low-frequency and high-frequency subimages LL_2_ and HH_2_, the pattern of each segment is analyzed and each segment is characterized by 2 feature vectors: one of length 3 extracted from LL_2_ containing the mean, mean absolute deviation (MAD), and skewness and another of length 2 extracted from HH_2_, containing the skewness and variance. In each subimage, the Euclidean distance between each pair of feature vectors is computed and a list consisting of the calculated distances is created and then ordered in increasing order. A pair of segments is marked as tampered if the distance appears in position *p* in one list and in position *p* + *t* in the other, *t* ranging from 0 to 3. Recently, in [83], the image is first segmented into objects using Chan Veses level set technique and, for each object, shape features are extracted. Then the Euclidean distance is used as similarity measure to compare shape signatures.

### 3.2. Subimage-Based Approaches

To detect copy-move forgeries, some methods partition the image into subimages that are most of the time of the same size. In [45], the low-frequency subband (of the wavelet decomposition of the input image) is first divided into four nonoverlapping subimages. The phase correlation between every pair of subimages is calculated to evaluate the spatial offset between the copied region and the pasted one. Then, the location of the forgery is done by shifting the input image in line with the obtained offset and subtracting this shifted image from the input image. the method is fairly robust to many types of postprocessing but fails in case the copy-move regions lie in more than one subimage. Authors in [84] proposed an approach to improve the one presented in [45] that can successfully detect the forgery even in case the copy-move regions do not lie in the same subimage. They divided the DWT-reduced image into nine subimages by using crossing shadow division. In [8], the grayscale representation Ig of the tested image is circularly shifted by a certain fraction of its size and the resulting image is horizontally (resp., vertically) divided into 2 parts of equal size. Then, the phase correlation of the 2 horizontal (resp., vertical) parts is computed. Next, the horizontal (resp., vertical) offset is computed and both offsets are used to calculate the shifted version of the image Is. Finally, Is is subtracted from Ig. This algorithm is robust to blurring and additive noise. Authors in [85] proposed an expanding block algorithm in which comparison is done directly on blocks rather than on features extracted from them as it is the case for other approaches. The input image is first divided into overlapping small blocks and, for each block, its average gray value is computed as the dominant feature. Blocks are sorted based on their respective dominant feature and then, as evenly as possible, grouped together after eliminating all block pairs that are extremely different in terms of their dominant feature. Finally, statistical hypothesis test is used to perform comparisons in each group.

### 3.3. Other Approaches

In this section, approaches that are partition-based but neither follow the pipeline described in Figure 3 nor fall in any of the categories indicated earlier are presented.

In [86], the target was the enhancement of the performance of copy-move forgeries detection scheme. This was done by porting onto the Graphic Processing Units most of the steps that can use data-parallel computing. By doing so, the performance was improved compared to other approaches and this without deterioration of the detection rate. In [87], the input image is decomposed in its bit-plane representation and a bit blocks encoding procedure is applied with an ASCII code for each bit-plane and each block. Then the resulting sequences of strings are analyzed to search for duplicated areas. The method produces a high accuracy in reasonable time but fails in case of scaling and/or rotation of the copied region and is not applicable to JPEG images. In [20], the suspicious image is first divided into nonoverlapping square *m* × *m* blocks that are represented by vectors of length *m* ^2^. Then, a local dimension estimation algorithm, for example, the k-NN algorithm, is applied on each block representation to get its local dimension estimation and the results are stored as columns of a matrix. This is followed by a neighborhood smoothing procedure on the obtained matrix enabling to segment the suspicious image into different regions based on the final local dimension estimator. A region here is defined as a group of blocks that share the common intrinsic dimension estimator. Once the segmentation is done, the copy-move forgery detection can be carried out by first dividing each texture region into overlapping square blocks of *m* × *m* pixels. Then, each block is represented as a vector of length *m* ^2^ and, for every pair of vectors within each texture region, the absolute value of their difference vector is computed. For each resulting vector, the local dimension estimation is obtained for each minimum; the corresponding blocks are marked as possible source and target blocks. The copy-move forgery central block minimum is determined based on the principle that the minimum difference between the source and target blocks is found in the center of the target region since, most of the time, the boundary of the copied region is retouched more than the central part within the target region. Once the central block is determined, the source and target regions are gradually extended by including the adjacent blocks with the increasing local dimension estimators of the difference vectors. Authors in [88, 89] decomposed the input image into approximation (LL_1_) and detail (HH_1_) subbands using dyadic wavelet transform (DyWT).Then, they divided each subband into overlapping blocks and computed the Euclidian distance between every pair of blocks. For each subband, an array constituted by the calculated distances is built and sorted in ascending order for LL_1_ subband and in descending order for HH_1_ subband. Then, they measured the similarity between blocks, using the Euclidian distance between them. The idea used is that a pair of duplicated blocks should exhibit a high similarity in the LL_1_ subband while exhibiting a high dissimilarity in the HH_1_ subband. Then, a pair is detected as forged if the distance between the corresponding blocks shows up in both lists at approximately the same location. The method is shift-invariant. In [90], the approach in [88, 89] is improved by applying DCT on blocks and comparing blocks pairs using the Euclidean distance of discrete cosine transformed blocks instead of the Euclidean distance of raw blocks. Let us note that only 30 DC coefficients are used. In [37], the input RGB color image is converted into the YCbCr chrominance space and a steerable pyramid transform (SPT) is applied on each channel of the YCbCR image to transform it into multiple subbands of different orientations and scales. On each SPT subband, local binary pattern (LBP) is applied and, for each subband, a feature vector is extracted as the normalized LBP histogram. Further, features are selected using two different methods for data dimensionality reduction and the classification of the image as copy-move tampered or authentic image is done using the support vector machine (SVM). This method aims at classifying the image without localizing the forged areas in the image in case the image is classified as tampered. In [91], the input image is first divided into nonoverlapping rectangular blocks. Then the mutual information of each block and the underlying image region is computed assuming that unforged regions will have zeros values while copy move forged regions will have some finite values. The method is robust to illumination changes but cannot localize the forged area. In [92], a phase correlation method based on polar expansion and adaptive band limitation is proposed for copy-move forgery detection. The technique starts by computing the Fourier transform of the polar expansion on overlapping circular windows pair, and then an adaptive band limitation procedure is implemented to obtain a correlation matrix having a peak that is adequately enhanced. After estimating the rotation angle of the forged region, a searching algorithm in the sense of seed filling is executed to display the entire duplicated area. The method is robust to rotation, blurring, illumination changes, and JPEG compression. In [93, 94], the method works for a manipulation performed on JPEG and the target image is also JPEG. As JPEG is one of the most frequently used format by cameras, the method is quite useful. Their approach utilizes the mismatch of information of block artifact grid as clue of copy paste forgery. A DCT grid is made of the horizontal lines and the vertical lines that partition an image into blocks and a block artifact grid (BAG) is the grid embedded in an image where block artifact appears. The DCT grid and BAG match together in nonforged images. Authors describe a BAG marking algorithm. BAG marking is even clear for JPEG compressed images of lower quality.

## 4. Conclusion

A concise survey on the partition-based copy-move forgery detection methods was presented that may help researchers to seek new concepts and provide new solutions to the challenges in the field. An attempt has been made to clearly present each step of the different existing techniques. Each of the techniques presented here has some drawbacks and, consequently, there is still more work to be done to perfect them. Some of the main challenges are the reduction of the computational complexity, an increased accuracy rate versus a decreased false detection rate, and the robustness to various transformations (rotation, noise addition, translation, scaling, lossy compression, blurring, flipping, edge sharpening, change of intensity, reflection, and contrast changes) regardless of their parameters (rotation angle, signal to noise ratio, shift vector, scaling factor, JPEG compression quality factor, etc.). There is also a great challenge to chose the most appropriate threshold values in the feature matching and forgery detection steps. Most of the time, the choice of the threshold values is not automated as it might vary with some characteristics of the input image such as its size. Consequently, it is important for each technique to clearly define the selection procedure of the optimal threshold values by identifying the images characteristics which might affect them and formally define how they relate to the said thresholds. In order to attempt to meet the above-mentioned challenges, there is a need to define a clear performance analysis methodology through the implementation of a validation framework. Furthermore, as new digital image capturing devices and image manipulation techniques continue to emerge, it is important to identify the detection techniques that have become invalid.
